# Aqua­(1,10-phenanthroline-κ^2^ *N*,*N*′)bis­(trimethyl­acetato)-κ^2^ *O*,*O*′;κ*O*-cobalt(II)

**DOI:** 10.1107/S1600536809027251

**Published:** 2009-07-29

**Authors:** Xiao-Dan Chen, Hong-Xian Chen, Zhong-Shu Li, Huai-Hong Zhang, Bai-Wang Sun

**Affiliations:** a Ordered Matter Science Research Center, College of Chemistry and Chemical Engineering, Southeast University, Nanjing 210096, People’s Republic of China; b Department of Chemistry, Key Laboratory of Medicinal Chemistry for Natural Resources, Ministry of Education, Yunnan University, Kunming 650091, People’s Republic of China

## Abstract

In the title compound, [Co(C_5_H_9_O_2_)_2_(C_12_H_8_N_2_)(H_2_O)], the Co^II^ atom is coordinated in a distorted octahedral environment by three carboxyl O atoms of two trimethyl­acetate ligands, one aqua O atom and two N atoms from 1,10-phen­anthroline. The crystal structure is stabilized by O—H⋯O hydrogen bonds and π–π stacking inter­actions [inter­planar distance between inter­digitating 1,10-phenanthroline ligands = 3.378 (2) Å].

## Related literature

For π-π stacking inter­actions, see: Mizutani *et al.* (1999 ▶); Sugimori *et al.* (1997 ▶). For the [CoN_2_O_4_] octahedral coordin­ation, see: Zheng *et al.* (2002 ▶).
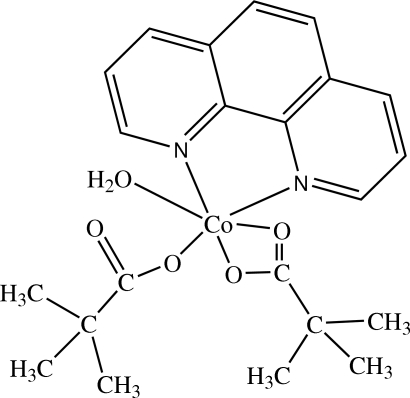


## Experimental

### 

#### Crystal data

[Co(C_5_H_9_O_2_)_2_(C_12_H_8_N_2_)(H_2_O)]
*M* *_r_* = 459.39
Triclinic, 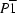

*a* = 10.905 (2) Å
*b* = 11.345 (2) Å
*c* = 11.476 (5) Å
α = 68.100 (6)°
β = 64.560 (5)°
γ = 63.230 (6)°
*V* = 1116.0 (6) Å^3^
*Z* = 2
Mo *K*α radiation
μ = 0.80 mm^−1^
*T* = 293 K
0.27 × 0.20 × 0.20 mm

#### Data collection

Rigaku SCXmini diffractometer
Absorption correction: multi-scan (*CrystalClear*; Rigaku/MSC, 2005 ▶) *T*_min_ = 0.831, *T*_max_ = 0.862
11372 measured reflections
5047 independent reflections
4271 reflections with *I* > 2σ(*I*)
*R*_int_ = 0.026

#### Refinement

*R*[*F* ^2^ > 2σ(*F* ^2^)] = 0.041
*wR*(*F* ^2^) = 0.103
*S* = 1.05
5047 reflections
271 parameters
H-atom parameters constrained
Δρ_max_ = 0.37 e Å^−3^
Δρ_min_ = −0.52 e Å^−3^

### 

Data collection: *CrystalClear* (Rigaku, 2005 ▶); cell refinement: *CrystalClear*; data reduction: *CrystalClear*; program(s) used to solve structure: *SHELXS97* (Sheldrick, 2008 ▶); program(s) used to refine structure: *SHELXL97* (Sheldrick, 2008 ▶); molecular graphics: *SHELXTL* (Sheldrick, 2008 ▶); software used to prepare material for publication: *SHELXTL*.

## Supplementary Material

Crystal structure: contains datablocks I, global. DOI: 10.1107/S1600536809027251/at2827sup1.cif

Structure factors: contains datablocks I. DOI: 10.1107/S1600536809027251/at2827Isup2.hkl

Additional supplementary materials:  crystallographic information; 3D view; checkCIF report

## Figures and Tables

**Table:** 

Co1—O2	2.0436 (15)
Co1—O1	2.0570 (15)
Co1—N1	2.1068 (17)
Co1—O5	2.1499 (16)
Co1—N2	2.1653 (18)
Co1—O4	2.2154 (16)

**Table:** 

O2—Co1—O1	90.02 (6)
O2—Co1—N1	91.44 (7)
O1—Co1—N1	108.97 (7)
O2—Co1—O5	93.03 (7)
O1—Co1—O5	155.63 (6)
N1—Co1—O5	95.13 (6)
O2—Co1—N2	167.76 (7)
O1—Co1—N2	88.59 (7)
N1—Co1—N2	77.54 (7)
O5—Co1—N2	93.25 (7)
O2—Co1—O4	92.99 (7)
O1—Co1—O4	96.07 (6)
N1—Co1—O4	154.56 (6)
O5—Co1—O4	59.64 (6)
N2—Co1—O4	99.25 (7)

**Table 2:** Hydrogen-bond geometry (Å, °)

*D*—H⋯*A*	*D*—H	H⋯*A*	*D*⋯*A*	*D*—H⋯*A*
O1—H1*A*⋯O3	0.93	1.84	2.584 (2)	135
O1—H1*C*⋯O4^i^	0.93	2.11	2.721 (2)	122

Symmetry code: (i) 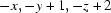.

**Fig. 1.**
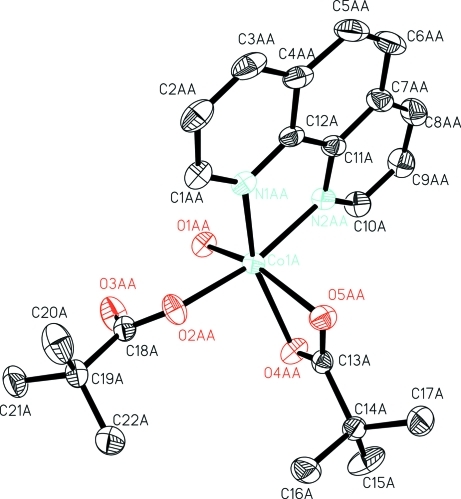
The molecular structure of the title molecule, with the atom-numbering scheme. Displacement ellipsoids are drawn at the 30% probability level. All hydrogen atoms are omitted for clarity.

**Fig. 2.**
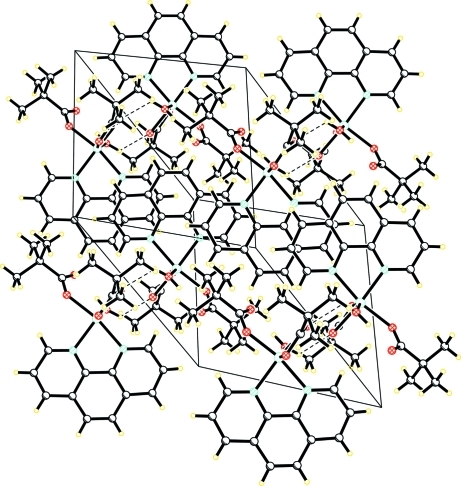
Crystal packing of the compound (I). Hydrogen bonds are shown as dashed lines.

## supplementary crystallographic information

### Comment

The crystal's center atom Co^II^ is coordinated by three carboxyl O atoms of two trimethylacetic acids, one aqua O atom and two N atoms from 1,10-phenanthroline (phen), what results in significantly distorted [CoN_2_O_4_] octahedral coordination (Zheng, *et al.*, 2002). As illustrated in Fig. 1, Co—O bond distances to the chelating carboxyl O atoms are 2.215 and 2.150 Å, substantially longer than those of 2.057, and 2.044 Å to the aqua O atom and the monodentates carboxyl O atom; Co—N bond distance are 2.107 and 2.165 Å. The chelating phen ligand exhibits nearly perfect coplanarity. Coordinating H_2_O molecules donate hydrogen atoms to the noncoordinating and coordinating carboxyl O atoms of different acid ligands to form strong intra- and inter-molecular hydrogen bonds with H···O = 1.84, 2.11 Å and O–H···O = 135, 122°. Due to such bonds the centrosymmetric dimmers are connected into one-dimensional chains (Fig.2).

Interdigitation of the chelating phen ligands of adjacent chains leads to formation of two-dimensional layers (Fig. 2). Interplanar distances between interdigitating phen ligands are 3.523 Å on average, suggesting significant intermolecular π-π stacking interactions (Mizutani, *et al.*, 1999; Sugimori *et al.*, 1997).

### Experimental

CoCO_3_ 100.1 mg (1 mmol) was added to a solution of 180.2 mg (1 mmol) 1,10-phenanthroline and 102.1 mg(1 mmol) trimethylacetic acids dissolved in 20 ml CH_3_OH—H_2_O (1:1 *v*/*v*) and stirred untile complete dissolution occurred, then, filtered the solution, laid in a peace environment. After a few days, colourless well shaped single crystals in the form of prisms deposited in the mother liquid. They were separated off, washed with cold ethanol and dried in air at room temperature.

### Refinement

H atoms were placed in geometrical positions and refined using a riding model, with O—H = 0.93 Å, C—H = 0.93 - 0.96 Å and *U*_iso_(H) = 1.2 or 1.5*U*_eq_(C, O). The water H atoms were located from the difference map and refined freely.

### Crystal data

**Table:**

[Co(C_5_H_9_O_2_)_2_(C_12_H_8_N_2_)(H_2_O)]	*Z* = 2
*M**_r_* = 459.39	*F*(000) = 482
Triclinic, *P*1	*D*_x_ = 1.367 Mg m^−^^3^
Hall symbol: -P 1	Mo *K*α radiation, λ = 0.71073 Å
*a* = 10.905 (2) Å	Cell parameters from 3476 reflections
*b* = 11.345 (2) Å	θ = 2.3–27.5°
*c* = 11.476 (5) Å	µ = 0.80 mm^−^^1^
α = 68.100 (6)°	*T* = 293 K
β = 64.560 (5)°	Prism, colourless
γ = 63.230 (6)°	0.27 × 0.20 × 0.20 mm
*V* = 1116.0 (6) Å^3^	

### Data collection

**Table:**

Rigaku SCXmini diffractometer	5047 independent reflections
Radiation source: fine-focus sealed tube	4271 reflections with *I* > 2σ(*I*)
graphite	*R*_int_ = 0.026
Detector resolution: 13.6612 pixels mm^-1^	θ_max_ = 27.4°, θ_min_ = 2.4°
Thin–slice ω scans	*h* = −14→14
Absorption correction: multi-scan (*CrystalClear*; Rigaku/MSC, 2005)	*k* = −14→14
*T*_min_ = 0.831, *T*_max_ = 0.862	*l* = −14→14
11372 measured reflections	

### Refinement

**Table:**

Refinement on *F*^2^	Primary atom site location: structure-invariant direct methods
Least-squares matrix: full	Secondary atom site location: difference Fourier map
*R*[*F*^2^ > 2σ(*F*^2^)] = 0.041	Hydrogen site location: inferred from neighbouring sites
*wR*(*F*^2^) = 0.103	H-atom parameters constrained
*S* = 1.05	*w* = 1/[σ^2^(*F*_o_^2^) + (0.0476*P*)^2^ + 0.3479*P*] where *P* = (*F*_o_^2^ + 2*F*_c_^2^)/3
5047 reflections	(Δ/σ)_max_ = 0.001
271 parameters	Δρ_max_ = 0.37 e Å^−^^3^
0 restraints	Δρ_min_ = −0.52 e Å^−^^3^

### Special details

**Table:**

Geometry. All e.s.d.'s (except the e.s.d. in the dihedral angle between two l.s. planes) are estimated using the full covariance matrix. The cell e.s.d.'s are taken into account individually in the estimation of e.s.d.'s in distances, angles and torsion angles; correlations between e.s.d.'s in cell parameters are only used when they are defined by crystal symmetry. An approximate (isotropic) treatment of cell e.s.d.'s is used for estimating e.s.d.'s involving l.s. planes.
Refinement. Refinement of *F*^2^ against ALL reflections. The weighted *R*-factor *wR* and goodness of fit *S* are based on *F*^2^, conventional *R*-factors *R* are based on *F*, with *F* set to zero for negative *F*^2^. The threshold expression of *F*^2^ > σ(*F*^2^) is used only for calculating *R*-factors(gt) *etc*. and is not relevant to the choice of reflections for refinement. *R*-factors based on *F*^2^ are statistically about twice as large as those based on *F*, and *R*- factors based on ALL data will be even larger.

### Fractional atomic coordinates and isotropic or equivalent isotropic displacement parameters (Å^2^)

**Table:**

	*x*	*y*	*z*	*U*_iso_*/*U*_eq_	
Co1	0.26826 (3)	0.38218 (3)	0.84914 (3)	0.03513 (10)	
C1	0.5399 (2)	0.3266 (2)	0.6055 (2)	0.0460 (5)	
H1B	0.4995	0.2767	0.5937	0.055*	
C2	0.6762 (3)	0.3305 (3)	0.5189 (2)	0.0583 (7)	
H2A	0.7241	0.2858	0.4498	0.070*	
C3	0.7377 (3)	0.4008 (3)	0.5370 (2)	0.0604 (7)	
H3A	0.8289	0.4030	0.4810	0.073*	
C4	0.6637 (2)	0.4695 (2)	0.6398 (2)	0.0507 (6)	
C5	0.7182 (3)	0.5469 (3)	0.6670 (3)	0.0662 (8)	
H5A	0.8110	0.5488	0.6169	0.079*	
C6	0.6393 (3)	0.6162 (3)	0.7623 (3)	0.0661 (8)	
H6A	0.6783	0.6652	0.7771	0.079*	
C7	0.4955 (3)	0.6168 (2)	0.8425 (3)	0.0514 (6)	
C8	0.4059 (3)	0.6901 (2)	0.9420 (3)	0.0620 (7)	
H8A	0.4376	0.7438	0.9590	0.074*	
C9	0.2720 (3)	0.6818 (3)	1.0133 (3)	0.0624 (7)	
H9A	0.2107	0.7311	1.0785	0.075*	
C10	0.2271 (3)	0.5989 (2)	0.9882 (2)	0.0527 (6)	
H10A	0.1354	0.5940	1.0386	0.063*	
C11	0.4410 (2)	0.5369 (2)	0.8231 (2)	0.0404 (5)	
C12	0.5258 (2)	0.4631 (2)	0.7205 (2)	0.0389 (5)	
C13	0.2435 (2)	0.2123 (2)	1.0675 (2)	0.0367 (4)	
C14	0.2332 (2)	0.0909 (2)	1.1843 (2)	0.0420 (5)	
C15	0.0878 (3)	0.1255 (3)	1.2913 (3)	0.0783 (10)	
H15A	0.0116	0.1560	1.2545	0.117*	
H15B	0.0772	0.1959	1.3258	0.117*	
H15C	0.0825	0.0465	1.3614	0.117*	
C16	0.2528 (3)	−0.0223 (3)	1.1296 (3)	0.0612 (7)	
H16A	0.1760	0.0058	1.0945	0.092*	
H16B	0.2509	−0.1020	1.1993	0.092*	
H16C	0.3441	−0.0418	1.0606	0.092*	
C17	0.3577 (4)	0.0419 (3)	1.2395 (3)	0.0760 (9)	
H17A	0.3506	−0.0343	1.3127	0.114*	
H17B	0.3519	0.1137	1.2688	0.114*	
H17C	0.4484	0.0155	1.1715	0.114*	
C18	0.1598 (2)	0.2719 (2)	0.7243 (2)	0.0408 (5)	
C19	0.1761 (3)	0.1485 (2)	0.6869 (2)	0.0465 (5)	
C20	0.3296 (3)	0.0940 (3)	0.5955 (3)	0.0764 (9)	
H20A	0.3404	0.0165	0.5717	0.115*	
H20B	0.3977	0.0684	0.6403	0.115*	
H20C	0.3474	0.1630	0.5170	0.115*	
C21	0.0678 (3)	0.1857 (3)	0.6171 (3)	0.0674 (7)	
H21A	0.0799	0.1069	0.5945	0.101*	
H21B	0.0841	0.2544	0.5380	0.101*	
H21C	−0.0288	0.2192	0.6749	0.101*	
C22	0.1490 (4)	0.0425 (3)	0.8136 (3)	0.0786 (9)	
H22A	0.1585	−0.0366	0.7933	0.118*	
H22B	0.0530	0.0781	0.8714	0.118*	
H22C	0.2183	0.0187	0.8564	0.118*	
O1	0.10121 (15)	0.53873 (15)	0.78736 (15)	0.0450 (4)	
H1A	0.0591	0.5256	0.7400	0.054*	
H1C	0.0678	0.6226	0.8068	0.054*	
O2	0.25728 (16)	0.25900 (16)	0.76443 (17)	0.0490 (4)	
O3	0.05280 (19)	0.37779 (19)	0.7150 (2)	0.0682 (5)	
O4	0.13089 (16)	0.30786 (15)	1.04733 (15)	0.0483 (4)	
O5	0.36512 (16)	0.21463 (16)	0.98721 (16)	0.0485 (4)	
N1	0.46630 (18)	0.39072 (17)	0.70320 (17)	0.0386 (4)	
N2	0.30790 (19)	0.52688 (17)	0.89663 (18)	0.0407 (4)	

### Atomic displacement parameters (Å^2^)

**Table:**

	*U*^11^	*U*^22^	*U*^33^	*U*^12^	*U*^13^	*U*^23^
Co1	0.02998 (15)	0.03444 (16)	0.03732 (16)	−0.00956 (11)	−0.00698 (11)	−0.01055 (11)
C1	0.0433 (12)	0.0422 (12)	0.0396 (11)	−0.0056 (10)	−0.0117 (10)	−0.0092 (9)
C2	0.0458 (13)	0.0608 (15)	0.0377 (12)	−0.0037 (12)	−0.0035 (11)	−0.0094 (11)
C3	0.0386 (12)	0.0688 (17)	0.0433 (13)	−0.0161 (12)	−0.0033 (11)	0.0045 (12)
C4	0.0385 (11)	0.0526 (13)	0.0453 (13)	−0.0193 (10)	−0.0116 (10)	0.0077 (11)
C5	0.0516 (15)	0.0687 (18)	0.0726 (18)	−0.0381 (14)	−0.0156 (14)	0.0069 (15)
C6	0.0641 (17)	0.0579 (16)	0.087 (2)	−0.0393 (14)	−0.0289 (16)	0.0018 (15)
C7	0.0579 (14)	0.0364 (11)	0.0643 (15)	−0.0216 (11)	−0.0274 (12)	0.0004 (11)
C8	0.0818 (19)	0.0400 (13)	0.0790 (19)	−0.0234 (13)	−0.0383 (16)	−0.0101 (13)
C9	0.0763 (18)	0.0443 (13)	0.0698 (17)	−0.0115 (13)	−0.0252 (15)	−0.0252 (13)
C10	0.0519 (13)	0.0469 (13)	0.0538 (14)	−0.0129 (11)	−0.0091 (11)	−0.0198 (11)
C11	0.0400 (11)	0.0336 (10)	0.0444 (12)	−0.0139 (9)	−0.0144 (9)	−0.0025 (9)
C12	0.0356 (10)	0.0356 (10)	0.0373 (11)	−0.0139 (9)	−0.0120 (9)	0.0029 (8)
C13	0.0407 (11)	0.0344 (10)	0.0362 (10)	−0.0099 (9)	−0.0124 (9)	−0.0126 (8)
C14	0.0510 (13)	0.0345 (10)	0.0403 (11)	−0.0128 (9)	−0.0155 (10)	−0.0093 (9)
C15	0.085 (2)	0.0597 (17)	0.0449 (15)	−0.0168 (16)	0.0033 (15)	−0.0036 (13)
C16	0.0826 (19)	0.0495 (14)	0.0597 (15)	−0.0311 (14)	−0.0184 (14)	−0.0150 (12)
C17	0.108 (2)	0.0553 (16)	0.087 (2)	−0.0338 (17)	−0.067 (2)	0.0124 (15)
C18	0.0323 (10)	0.0515 (12)	0.0380 (11)	−0.0126 (9)	−0.0069 (9)	−0.0169 (10)
C19	0.0456 (12)	0.0543 (13)	0.0463 (12)	−0.0159 (10)	−0.0159 (10)	−0.0181 (11)
C20	0.0578 (16)	0.088 (2)	0.091 (2)	−0.0096 (15)	−0.0136 (16)	−0.0597 (19)
C21	0.0757 (19)	0.0776 (19)	0.0733 (18)	−0.0313 (16)	−0.0385 (16)	−0.0182 (15)
C22	0.120 (3)	0.0628 (18)	0.074 (2)	−0.0452 (19)	−0.047 (2)	−0.0005 (15)
O1	0.0391 (8)	0.0383 (8)	0.0444 (8)	−0.0016 (6)	−0.0120 (7)	−0.0116 (7)
O2	0.0395 (8)	0.0492 (9)	0.0671 (10)	−0.0054 (7)	−0.0226 (8)	−0.0275 (8)
O3	0.0481 (10)	0.0667 (12)	0.0999 (15)	0.0029 (9)	−0.0371 (10)	−0.0413 (11)
O4	0.0412 (8)	0.0419 (8)	0.0395 (8)	−0.0022 (7)	−0.0075 (7)	−0.0078 (7)
O5	0.0393 (8)	0.0500 (9)	0.0503 (9)	−0.0158 (7)	−0.0147 (7)	−0.0041 (7)
N1	0.0352 (9)	0.0361 (9)	0.0350 (9)	−0.0083 (7)	−0.0084 (7)	−0.0065 (7)
N2	0.0388 (9)	0.0367 (9)	0.0436 (10)	−0.0125 (8)	−0.0087 (8)	−0.0118 (8)

### Geometric parameters (Å, °)

**Table:**

Co1—O2	2.0436 (15)	C13—C14	1.529 (3)
Co1—O1	2.0570 (15)	C14—C15	1.518 (3)
Co1—N1	2.1068 (17)	C14—C16	1.525 (3)
Co1—O5	2.1499 (16)	C14—C17	1.533 (3)
Co1—N2	2.1653 (18)	C15—H15A	0.9600
Co1—O4	2.2154 (16)	C15—H15B	0.9600
C1—N1	1.320 (3)	C15—H15C	0.9600
C1—C2	1.399 (3)	C16—H16A	0.9600
C1—H1B	0.9300	C16—H16B	0.9600
C2—C3	1.361 (4)	C16—H16C	0.9600
C2—H2A	0.9300	C17—H17A	0.9600
C3—C4	1.398 (4)	C17—H17B	0.9600
C3—H3A	0.9300	C17—H17C	0.9600
C4—C12	1.406 (3)	C18—O3	1.251 (3)
C4—C5	1.433 (4)	C18—O3	1.251 (3)
C5—C6	1.334 (4)	C18—O2	1.263 (2)
C5—H5A	0.9300	C18—C19	1.528 (3)
C6—C7	1.436 (4)	C19—C22	1.515 (4)
C6—H6A	0.9300	C19—C21	1.525 (3)
C7—C8	1.403 (4)	C19—C20	1.526 (4)
C7—C11	1.404 (3)	C20—H20A	0.9600
C8—C9	1.360 (4)	C20—H20B	0.9600
C8—H8A	0.9300	C20—H20C	0.9600
C9—C10	1.395 (3)	C21—H21A	0.9600
C9—H9A	0.9300	C21—H21B	0.9600
C10—N2	1.321 (3)	C21—H21C	0.9600
C10—H10A	0.9300	C22—H22A	0.9600
C11—N2	1.363 (3)	C22—H22B	0.9600
C11—C12	1.430 (3)	C22—H22C	0.9600
C12—N1	1.358 (3)	O1—H1A	0.9300
C13—O5	1.254 (2)	O1—H1C	0.9300
C13—O4	1.263 (2)	O3—O3	0.000 (5)
			
O2—Co1—O1	90.02 (6)	C14—C15—H15B	109.5
O2—Co1—N1	91.44 (7)	H15A—C15—H15B	109.5
O1—Co1—N1	108.97 (7)	C14—C15—H15C	109.5
O2—Co1—O5	93.03 (7)	H15A—C15—H15C	109.5
O1—Co1—O5	155.63 (6)	H15B—C15—H15C	109.5
N1—Co1—O5	95.13 (6)	C14—C16—H16A	109.5
O2—Co1—N2	167.76 (7)	C14—C16—H16B	109.5
O1—Co1—N2	88.59 (7)	H16A—C16—H16B	109.5
N1—Co1—N2	77.54 (7)	C14—C16—H16C	109.5
O5—Co1—N2	93.25 (7)	H16A—C16—H16C	109.5
O2—Co1—O4	92.99 (7)	H16B—C16—H16C	109.5
O1—Co1—O4	96.07 (6)	C14—C17—H17A	109.5
N1—Co1—O4	154.56 (6)	C14—C17—H17B	109.5
O5—Co1—O4	59.64 (6)	H17A—C17—H17B	109.5
N2—Co1—O4	99.25 (7)	C14—C17—H17C	109.5
N1—C1—C2	122.7 (2)	H17A—C17—H17C	109.5
N1—C1—H1B	118.7	H17B—C17—H17C	109.5
C2—C1—H1B	118.7	O3—C18—O3	0.0 (2)
C3—C2—C1	119.1 (2)	O3—C18—O2	123.9 (2)
C3—C2—H2A	120.4	O3—C18—O2	123.9 (2)
C1—C2—H2A	120.4	O3—C18—C19	119.52 (19)
C2—C3—C4	120.0 (2)	O3—C18—C19	119.52 (19)
C2—C3—H3A	120.0	O2—C18—C19	116.59 (19)
C4—C3—H3A	120.0	C22—C19—C21	109.9 (2)
C3—C4—C12	117.3 (2)	C22—C19—C20	109.9 (3)
C3—C4—C5	124.4 (2)	C21—C19—C20	109.5 (2)
C12—C4—C5	118.3 (2)	C22—C19—C18	107.5 (2)
C6—C5—C4	121.6 (2)	C21—C19—C18	110.8 (2)
C6—C5—H5A	119.2	C20—C19—C18	109.2 (2)
C4—C5—H5A	119.2	C19—C20—H20A	109.5
C5—C6—C7	121.5 (3)	C19—C20—H20B	109.5
C5—C6—H6A	119.3	H20A—C20—H20B	109.5
C7—C6—H6A	119.3	C19—C20—H20C	109.5
C8—C7—C11	117.3 (2)	H20A—C20—H20C	109.5
C8—C7—C6	124.2 (2)	H20B—C20—H20C	109.5
C11—C7—C6	118.5 (2)	C19—C21—H21A	109.5
C9—C8—C7	119.2 (2)	C19—C21—H21B	109.5
C9—C8—H8A	120.4	H21A—C21—H21B	109.5
C7—C8—H8A	120.4	C19—C21—H21C	109.5
C8—C9—C10	119.7 (2)	H21A—C21—H21C	109.5
C8—C9—H9A	120.2	H21B—C21—H21C	109.5
C10—C9—H9A	120.2	C19—C22—H22A	109.5
N2—C10—C9	123.4 (2)	C19—C22—H22B	109.5
N2—C10—H10A	118.3	H22A—C22—H22B	109.5
C9—C10—H10A	118.3	C19—C22—H22C	109.5
N2—C11—C7	123.2 (2)	H22A—C22—H22C	109.5
N2—C11—C12	116.97 (18)	H22B—C22—H22C	109.5
C7—C11—C12	119.8 (2)	Co1—O1—H1A	120.0
N1—C12—C4	122.4 (2)	Co1—O1—H1C	120.0
N1—C12—C11	117.48 (18)	H1A—O1—H1C	120.0
C4—C12—C11	120.1 (2)	C18—O2—Co1	130.80 (14)
O5—C13—O4	119.26 (19)	O3—O3—C18	0(10)
O5—C13—C14	119.71 (19)	C13—O4—Co1	88.47 (12)
O4—C13—C14	120.97 (19)	C13—O5—Co1	91.69 (12)
C15—C14—C16	110.2 (2)	C1—N1—C12	118.54 (19)
C15—C14—C13	111.23 (19)	C1—N1—Co1	126.78 (16)
C16—C14—C13	106.70 (18)	C12—N1—Co1	114.51 (13)
C15—C14—C17	111.0 (2)	C10—N2—C11	117.2 (2)
C16—C14—C17	107.7 (2)	C10—N2—Co1	129.99 (16)
C13—C14—C17	109.8 (2)	C11—N2—Co1	112.71 (14)
C14—C15—H15A	109.5		
			
N1—C1—C2—C3	1.5 (4)	O2—C18—O3—O3	0.00 (14)
C1—C2—C3—C4	−1.0 (4)	C19—C18—O3—O3	0.00 (8)
C2—C3—C4—C12	−0.5 (3)	O5—C13—O4—Co1	9.51 (19)
C2—C3—C4—C5	−179.6 (2)	C14—C13—O4—Co1	−167.41 (17)
C3—C4—C5—C6	176.4 (3)	O2—Co1—O4—C13	86.16 (12)
C12—C4—C5—C6	−2.8 (4)	O1—Co1—O4—C13	176.50 (12)
C4—C5—C6—C7	0.0 (4)	N1—Co1—O4—C13	−13.5 (2)
C5—C6—C7—C8	−178.4 (3)	O5—Co1—O4—C13	−5.59 (11)
C5—C6—C7—C11	3.1 (4)	N2—Co1—O4—C13	−93.94 (13)
C11—C7—C8—C9	−0.5 (4)	O4—C13—O5—Co1	−9.8 (2)
C6—C7—C8—C9	−179.1 (3)	C14—C13—O5—Co1	167.15 (16)
C7—C8—C9—C10	1.1 (4)	O2—Co1—O5—C13	−86.06 (13)
C8—C9—C10—N2	−0.5 (4)	O1—Co1—O5—C13	10.7 (2)
C8—C7—C11—N2	−0.8 (3)	N1—Co1—O5—C13	−177.79 (12)
C6—C7—C11—N2	177.9 (2)	N2—Co1—O5—C13	104.45 (13)
C8—C7—C11—C12	178.2 (2)	O4—Co1—O5—C13	5.63 (11)
C6—C7—C11—C12	−3.2 (3)	C2—C1—N1—C12	−0.3 (3)
C3—C4—C12—N1	1.7 (3)	C2—C1—N1—Co1	−175.11 (16)
C5—C4—C12—N1	−179.1 (2)	C4—C12—N1—C1	−1.3 (3)
C3—C4—C12—C11	−176.7 (2)	C11—C12—N1—C1	177.10 (18)
C5—C4—C12—C11	2.5 (3)	C4—C12—N1—Co1	174.11 (16)
N2—C11—C12—N1	1.0 (3)	C11—C12—N1—Co1	−7.4 (2)
C7—C11—C12—N1	−178.02 (19)	O2—Co1—N1—C1	−2.54 (18)
N2—C11—C12—C4	179.46 (19)	O1—Co1—N1—C1	−93.05 (18)
C7—C11—C12—C4	0.5 (3)	O5—Co1—N1—C1	90.63 (18)
O5—C13—C14—C15	161.7 (2)	N2—Co1—N1—C1	−177.17 (18)
O4—C13—C14—C15	−21.4 (3)	O4—Co1—N1—C1	97.5 (2)
O5—C13—C14—C16	−78.0 (2)	O2—Co1—N1—C12	−177.56 (14)
O4—C13—C14—C16	98.9 (2)	O1—Co1—N1—C12	91.92 (14)
O5—C13—C14—C17	38.4 (3)	O5—Co1—N1—C12	−84.39 (14)
O4—C13—C14—C17	−144.6 (2)	N2—Co1—N1—C12	7.81 (13)
O3—C18—C19—C22	−109.2 (3)	O4—Co1—N1—C12	−77.5 (2)
O3—C18—C19—C22	−109.2 (3)	C9—C10—N2—C11	−0.8 (4)
O2—C18—C19—C22	70.2 (3)	C9—C10—N2—Co1	175.28 (19)
O3—C18—C19—C21	10.9 (3)	C7—C11—N2—C10	1.4 (3)
O3—C18—C19—C21	10.9 (3)	C12—C11—N2—C10	−177.5 (2)
O2—C18—C19—C21	−169.6 (2)	C7—C11—N2—Co1	−175.31 (17)
O3—C18—C19—C20	131.6 (3)	C12—C11—N2—Co1	5.7 (2)
O3—C18—C19—C20	131.6 (3)	O2—Co1—N2—C10	150.4 (3)
O2—C18—C19—C20	−49.0 (3)	O1—Co1—N2—C10	66.8 (2)
O3—C18—O2—Co1	9.3 (4)	N1—Co1—N2—C10	176.6 (2)
O3—C18—O2—Co1	9.3 (4)	O5—Co1—N2—C10	−88.9 (2)
C19—C18—O2—Co1	−170.10 (15)	O4—Co1—N2—C10	−29.1 (2)
O1—Co1—O2—C18	−15.6 (2)	O2—Co1—N2—C11	−33.4 (4)
N1—Co1—O2—C18	−124.6 (2)	O1—Co1—N2—C11	−116.99 (15)
O5—Co1—O2—C18	140.2 (2)	N1—Co1—N2—C11	−7.21 (14)
N2—Co1—O2—C18	−99.0 (3)	O5—Co1—N2—C11	87.33 (15)
O4—Co1—O2—C18	80.5 (2)	O4—Co1—N2—C11	147.09 (14)

### Hydrogen-bond geometry (Å, °)

**Table:**

*D*—H···*A*	*D*—H	H···*A*	*D*···*A*	*D*—H···*A*
O1—H1A···O3	0.93	1.84	2.584 (2)	135
O1—H1C···O4^i^	0.93	2.11	2.721 (2)	122

Symmetry codes:  (i) −*x*, −*y*+1, −*z*+2.
